# Symptoms and Otorhinolaryngological Sequalae in Long Covid

**DOI:** 10.1055/s-0045-1809026

**Published:** 2025-06-03

**Authors:** Alessandra Loli, João Victor Costa Müller, Eric Schneider de Azevedo, Regina Helena Garcia Martins

**Affiliations:** 1 Department of Ophthalmology, Otorhinolaryngology, and Head and Neck Surgery, Universidade Estadual Paulista Julio de Mesquita Filho, Botucatu Medical School, Unesp, São Paulo, SP, Brazil; 2 Department of Surgical Specialties and Anesthesiology, Universidade Estadual paulista Júlio de Mesquita Filho, Botucatu Medical School, Unesp, São Paulo, Brazil

**Keywords:** long covid, otorhinolaryngological sequelae, otorhinolaryngological symptoms

## Abstract

**Introduction:**

After the pandemic caused by the coronavirus, many patients have presented remaining otorhinolaryngological symptoms, but most of them are unknown to health professionals.

**Objectives:**

To characterize otorhinolaryngological symptoms and sequelae in hospitalized patients for Covid-19.

**Methods:**

We made a recall to patients hospitalized between April 2020 and April 2022 due to Covid-19. Demographic data, initial and remaining symptoms, days of hospitalization, intubation and/or tracheostomy, and vaccination data were collected.

**Results:**

845 patients were hospitalized, 441 died, 404 patients were contacted by telephone, but only 109 responded to the questionnaire about initial and remaining otorhinolaryngological symptoms after 1.5 to 2 years of illness, 59 men and 50 women, with an average age of 58.61 years (20 to 94). Two study groups were composed: G 1 (n- 44; with remaining symptoms) and G 2 (n- 65; without remaining symptoms). 81% of patients in G1 and 67% of patients in G2 had been hospitalized for up to 20 days. Intubation occurred in 17 patients (G1–7; G2–10). Seven patients underwent tracheostomy. The most prevalent initial and remaining otorhinolaryngological symptoms, respectively, were dyspnea (68.8%; 14.6%), cough (65.1%; 11.92%), nasal obstruction (47.7%; 5.58%), smell dysfunction (44%; 11%), taste dysfunction (42%; 4.58%). Vaccination was reported by 54 patients (G1–21; G2–34).

**Conclusions:**

Otorhinolaryngological symptoms were common in patients hospitalized for Covid-19, especially dyspnea, cough, nasal obstruction, smell, and taste dysfunction. Although there was a favorable long-term evolution, 40% of patients maintained permanent symptoms, such as smell and taste dysfunction and dizziness, unrelated to the vaccine doses.

## Introduction

Coronavirus disease 2019 (COVID-19) was a viral pandemic that emerged from the city of Wuhan, China, in December 2019 and quickly spread to the rest of the world. 1 The virus is transmitted from person to person through droplets and aerosols, as well as direct contact with the oral, nasal, and ocular mucous membranes. 2 The virus uses the angiotensin-converting enzyme 2 (ACE2) receptor to infect cells and cause tissue damage. The S protein (Spike) present on the surface of the viral capsule recognizes the ACE2 receptor and interacts with it, enabling the virus replication. It penetrates the mucous membranes of the nose, mouth, and eyes and can progress to the trachea, bronchi, and lungs. 2 Otorhinolaryngological symptoms such as cough, fever, expectoration, dyspnea, smell and taste dysfunction, rhinorrhea, and sore throat are common in patients with Covid-19. Among these, loss in the sense of smell (anosmia) (47.8% to 52%) and taste (ageusia) (43%) stand out. 3 4 5

With increased patient survival and a better understanding of the disease's progress, it was noted that some initial symptoms of the infection caused by COVID-19 persisted, becoming chronic. Covid disease encompasses three phases of signs and symptoms: (I) acute, persisting for up to 4 weeks; (II) subacute, from 4 to 12 weeks; and (III) post-COVID syndrome, lasting more than 12 weeks, known as long COVID. In the acute phase of the disease, symptoms of chemosensory dysfunction such as anosmia and ageusia appear. 6 7 Hyposmia may be present without any other systemic symptom, suggesting direct viral damage to the chemosensory system. Olfactory symptoms may begin suddenly or have a mild progression, persisting for days or years. 8 9

The same pathophysiology of viral neurotropism applies to the impact of coronavirus in the vestibulocochlear system, which is responsible for symptoms such as deafness, vertigo, and tinnitus. In addition, the virus directly invades the cochlear nerve or the membranous labyrinth of the inner ear. 10 Viral neurotropism can also compromise the vagus nerve, resulting in sudden or progressive dysphonia, secondary to paresis or paralysis of one or both vocal folds. 11 Oral lesions 12 and dysphagia 13 may also occur.

Our study is justified by the many symptoms related to COVID-19 and its different courses, in addition to the relevance of the topic for specialists, as well as the scarcity of substantial studies with long-term follow-up. The objective of this study was to characterize the otorhinolaryngological symptoms of patients admitted to a university hospital due to COVID, evaluate the course of these symptoms, identify possible sequelae, and correlate them with vaccination.

## Methods

Adult patients who had been admitted to our university hospital due to COVID-19 between April 2020 and April 2022 and who survived the infection were initially identified. The survivors were then contacted by telephone to collect data on age, gender, length of hospital stay, need for intubation and/or tracheostomy, vaccination, and number of doses. The patients were then actively questioned about having had specific otorhinolaryngological symptoms in the initial stages of the disease, as well as about symptoms that persisted to the day of the inquiry (between 1.5 and 2 years after the onset of the disease), namely, dyspnea, cough, nasal obstruction, rhinorrhea, smell and/or taste dysfunction, headache, pain or difficulty in swallowing, changes in salivation (dry mouth or sialorrhea), hoarseness/dysphonia, vertigo, tinnitus, otalgia, deafness, lesions in oral mucosa, sleep apnea, and facial paralysis. Other relevant symptoms reported by patients were also recorded. Based on the responses, two study groups were created: G1 (patients with symptoms remaining until the day of the interview) and G2 (patients with no remaining symptoms, considered completely free of otorhinolaryngological symptoms).

Exclusion criteria: patients with previous symptoms or history of treatment for otorhinolaryngological conditions before COVID-19, such as recurrent upper airway infections, chronic sinusitis, allergic rhinitis, previous otorhinolaryngological surgeries, history of deafness, chronic otitis, episodes of vertigo and tinnitus, patients diagnosed with obstructive sleep apnea syndrome, patients with previous facial paralysis, long-term dysphonic patients before the Covid infection, patients diagnosed with or undergoing treatment for oncological diseases in the head or neck, users of inhaled illicit drugs, and workers exposed to intense noise.

The data were arranged in tables and analyzed descriptively and numerically. For frequency analysis, the chi-square test was applied, considering a significance level of *p* < 0.05.

The project was approved by the Human Research Ethics Committee of the School of Medicine of Botucatu, under protocol number: 4899436 (CAAE: 50479421. 3. 0000. 5411).

## Results

During the study period, 845 adult patients were hospitalized due to Covid-19, of which 441 (52.2%) died, telephone contact was made to 404 patients, and 109 patients agreed to participate in the study. Two study groups were created: G1 (n-44; patients with remaining symptoms) and G2 (n-65; patients without remaining symptoms).

### Gender and Age

Table 1 shows the distribution of patients about gender and age group. A total of 59 (54.13%) patients were men and 50 (45.88%) were women. The average age was 58.61 years (ranging from 20 years to 94 years). The age group from 21 to 70 years concentrated the largest number of patients, both in G1 (79%) and G2 (73%). There was no statistical difference between the groups about gender or age.

**Table 1:** Sex and age range of both groups

Sex and Age range (years)	G1 (44) N(%)	G2 (65) N(%)	P*
Male	27(61.36)	32 (49.24))	0.293
Female	17(38.64)	33 (50.76)	
< 20	0	2 (3.08)	0.654
21 a 50	13 (29.55)	25(38.46)	0.4511
51 a 70	22 (50.00)	23(35.38)	O.186
>70	9(20.45)	15(23.08)	0.9284

* Chi-square test. P no significant (p > 0.05).

### Hospitalization Days

Table 2 presents information regarding the number of days in the hospital for both groups. Many patients in both groups remained hospitalized for less than 10 days. Both groups had similar lengths of stay, with no statistical difference.

**Table 2:** Days of hospitalization

Days of hospitalization	G1 N(%)	G2 N(%)	* *P* value
< 10	19 (43.18)	28(43.08)	1
10 a 20	17(38.63)	16(24.61)	0.176
21 a 30	2(4.55)	11(16.92)	0.097
>30	6(13.64)	10(15.39)	1

* Chi-square test. P no significant (p > 0.05).

### Days of Endotracheal Intubation

Endotracheal intubation was necessary in 17 patients (G1–7; G2–10). The minimum intubation time was one day and the maximum was 50 days. Tracheotomy was performed in 7 patients (G1–3; G2–4). Table 3 .

**Table 3:** Days of intubation

Days of intubation	G1(44) N(%)	G2(65) N(%)	* *P* value
< 7	0	3	0.3418
8 a 15	4	3	0.5363
16 a 30	1	2	1
>30	2	2	1

* Chi-square test. P no significant (p > 0.05).

### Initial and Remaining Otorhinolaryngological Symptoms

**Fig. 1:**
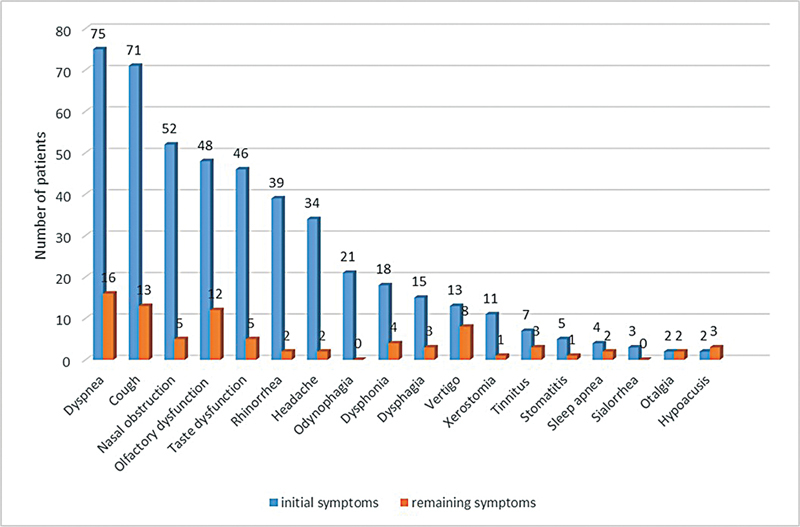
Initial and remaining otorhinolaryngological symptoms.

Table 4 shows the initial and remaining symptoms reported by the 109 patients. These symptoms decreased drastically over the years, however, some of them persisted in 44 patients (40%; G1). ( Table 4 and Fig. 1 ).

**Table 4:** Characterization of initial and remaining otorhinolaryngological symptoms

Symptoms	Initial symptoms N (%)	Remaining symptoms N(%)	* *P* value
Dyspnea	75 (68.8)	16 (14.68)	<0.0001*
Cough	71 (65.1)	13 (11.92)	<0.0001*
Nasal obstruction	52 (47.7)	5(4.58)	<0.0001*
Olfactory dysfunction	48 (44.0)	12 (11.01)	<0.0001*
Taste dysfunction	46 (42.2)	5 (4.58)	<0.0001*
Rhinorrhea	39 (35.8)	2 (1.83)	<0.0001*
Headage	34 (31.2)	2 (1.83)	<0.0001*
Odynophagia	21 (19.3)	0 (0)	<0.0001*
Dysphonia	18 (16.5)	4 (3.67)	0.0034*
Dysphagia	15 (13.7)	3 (2.75)	0.0067*
Vertigo	13 (11.9)	8 (7.34)	0.3585
Xerostomia	11 (10.1)	1 (0.92)	0.0076*
Tinnitus	7 (6.4)	3 (2.75)	0.3314
Stomatitis	5 (4.6)	1 (0.92)	0.6138
Sleep apnea	4 (3.7)	2 (1.83)	0.6789
Sialorrhea	3 (2.7)	0 (0.0)	0.2449
Otalgia	2 (1.8)	2 (1.83)	1
Hypoacusis	2 (1.8)	3 (2.75)	1

* Chi-square test. P significant <0.05 (*).

### Vaccination

Table 5 depicts the number of patients vaccinated, as well as the number of doses. A total of 54 patients (49.5%) had taken the vaccine before becoming ill (G1–20; G2–34), 27% of patients from G1 and 32% from G2 had received 2 or 3 doses. There was no relationship between the number of doses and the presence of otorhinolaryngological sequelae.

**Table 5:** Vaccine doses in both groups

Dose	G1 (n-44) N(%)	G2 (n-65) N(%)	* *P* value
1	6 (13.6)	12(18.45)	0.882
2	8(18.2)	11(16.92)	1
3	4(9.1)	10(15.38)	0.632
4	2(4.5)	1(1.54)	0.645
**Total**	**20(45.5)**	**34(52.3)**	

* Chi-square test. P no significant (p > 0.05).

## Discussion

In this study, most patients were between 21 and 70 years old (67%). Patients over 70 represented only 20% of the cases. There was a slight predilection for males (54% versus 46%), without statistical significance. Intubation was required in 17 patients (15.5%). Vaccination was reported by 54 patients (49.5%), of which 33 (30%) had received two or three doses. The effectiveness of vaccination has been questioned, and some publications highlight possible side effects of the vaccine such as tinnitus, vestibular changes, sensorineural hearing loss, facial paralysis, and epistaxis. 14 However, it should be noted that these correlations have not been confirmed until now.

Otorhinolaryngological symptoms in patients with Covid-19 are frequent, as also demonstrated by Qiu et al. 15 in a systematic review that included 16,478 patients. The authors highlighted, among the main symptoms, olfactory dysfunction (47%), sneezing (27%), nasal congestion (19%), expectoration (22%), sore throat (16%), rhinorrhea (14%), dyspnea (12%), and dizziness. (9%). These symptoms corroborate our results, in which dyspnea and cough stood out (68.8% and 65.1%) respectively, followed by nasal obstruction (47.7%), anosmia (44%), and ageusia (42.2%). Salepci et al. 16 interviewed 223 patients diagnosed with Covid-19 and the main otorhinolaryngological symptoms were anosmia (34.5%), and ageusia (31.8%), percentages slightly lower than those found in our study.

With viral attenuation and more effective vaccination, the survival rate of patients infected with the coronavirus has increased, enabling a better understanding of the course of the symptoms and sequelae of “post-covid-19 syndrome,” even though much about it is unknown until today. Xiong et al. 17 described respiratory, cardiovascular, psychosocial sequelae, and alopecia three months after hospital discharge of patients with Covid-19. In our study, 44 patients (40%) persisted with at least one symptom after 1.5 to 2 years, mainly dyspnea (14.6%), cough (11.92%), nasal obstruction (4.58%), anosmia (11%) and ageusia (4.6%), and vertigo (7.3%).

ENT symptoms tend to improve considerably after the acute phase of the disease, as evidenced in our results, however, abnormal smell, taste, balance, and dysphonia, are more resistant symptoms. Boscolo-Rizzo et al., 18 demonstrated the stages of recovery of smell and taste disorders in 168 patients with post-covid-19 symptoms such as symptoms present in the acute phase (n-119), after four weeks (n-64), after eight weeks (n-29), after six months (n-27) and after two years (n-14). According to these authors, in general, 28% of patients presented some remaining symptoms of Covid-19 after two years, a rate lower than that found by us (40%) in hospitalized patients.

The pathophysiology of olfactory disorders in Covid-19 includes direct toxic action of SARS-COV-2 on epithelial cells, via angiotensin-converting enzyme 2 (ACE2), expressed in the nasal ciliated epithelium and olfactory bulb; destruction of the olfactory nerve due to the neurotropic action of the virus; viral ascension through the cribriform plate of the olfactory bulb to the structures of the central nervous system, resulting in chronic dysfunction. Leukocytes and inflammatory cytokines were identified in the olfactory epithelium, responsible for the acute symptoms. The destruction of the structures of the olfactory bulb would be responsible for the typical symptoms, however, the exact course of olfactory disorders is still uncertain. 19 20 21

In an interesting review that evaluated the effects of smell and taste after Covid, Liao et al. 21 found an incidence of 33% to 85% for smell disorders and 64% to 88% for taste disorders. This large variation in percentages is due to the heterogeneity of data collection (questionnaires, objective tests, or intensity scales), as well as the profile of patients (outpatient or hospitalized). Studies with follow-up between three and 12 months indicated variable rates of symptom persistence, between 2.8% and 58%. Studies with longer follow-ups, over 12 months, recorded lower rates of symptoms, ranging from 3.1% to 25% for smell disorders and from 1.75% to 21.3%. % for taste disorders. In our study, our rates of persistence for both symptoms were 11% and 4.6%, respectively.

Otoneurological symptoms are also common in patients with Covid-19, such as conductive or sensorineural deafness, tinnitus, and vertigo, due to involvement of the inner ear. Viral neurotropism has been considered responsible for the symptoms of deafness and vertigo. The hematogenous route is also suggested, with hemoglobin as the virus vector, affecting all tissues, as it requires receptors for the angiotensin-converting enzyme 2 (ACE2), which is found in abundance in the brain, medulla oblongata, temporal lobe, where the auditory centers must be affected by inflammatory mediators. Furthermore, deoxygenation of erythrocytes may occur, culminating in hypoxia in the auditory centers. Another possibility would be ischemia of these centers, secondary to episodes of vasculitis and direct inflammatory effect on the brain caused by the SARS-CoV-2 spike protein, compromising the auditory centers and the inner ear through neuroinflammation, resulting in thrombotic and ischemic events and, consequently, local hypoxia. 10 22

Cochleovestibular symptoms are reported by patients in the acute, subacute, and chronic phases of the disease. Aydin et al. 22 demonstrated higher audiometric thresholds in post-Covid-19 patients than in control patients, especially for high frequencies, as well as changes in vestibular exams, indicating possible vestibular sequelae. A systematic review on neurological manifestations in Covid patients included 24 studies and highlighted the prevalence of dizziness (7%), changes in smell (19%), changes in taste (21%), hearing deficits (3%), and tinnitus (5%). 23 In our study, except for hearing deficit, all other symptoms were more relevant in the acute phase of the disease than those presented by these authors. Tan et al. 24 conducted a prospective study on cochleovestibular symptoms in patients who had Covid-19 for at least 30 days (26 patients with Covid-19 and 27 controls). An increase in audiometric thresholds was found in the covid group at frequencies of 4,000 Hz and 8,000 Hz, without changes in otoacoustic emissions. Significant differences were seen in vestibular tests between the groups, confirming the impairment of the cochleovestibular system by Covid-19. De Luca et al. 25 reported a prevalence of vertigo in post-Covid syndrome of 7.2%, a percentage close to that of our study.

Few studies presented results from post-Covid evaluations with follow-up longer than one year. According to the global analysis of the cumulative prevalence of “long Covid,” the rate of sequelae varies between 9% and 63%, much higher than that of any other virus. The extensive prevalence range depends on the duration of follow-up, hospitalization, comorbidities, smoking status, and patient age. O'Mahoney et al., 26 in a recent systematic review, identified sequelae in 52.6% of hospitalized patients, 34.4% of non-hospitalized patients, and 38% in both conditions (hospitalized and nom-hospitalized). Fatigue (28.4%; 34%; 25.2%), dyspnea (23.4%; 20.4%; 18.2%), and cough (10.2%; 6.5%; 10.6%) were the main remaining symptoms in the three groups, respectively. The most frequent otorhinolaryngological sequelae were anosmia (6.3%; 12.6%; 14.9%), ageusia (4.6%; 6.7%; 14.1), and nasal obstruction (4.1; 5 0.28; 9.9). Our rates of remaining olfactory symptoms were higher (11%), but for taste disorders, they were similar (4.6%). Some authors have studied the incidence of smell or taste disturbance in covid covariants (Delta, Alfa e Omicron) post-pandemic wave and observed a considerable decrease in these symptoms. 27

Arjun et al. 28 contacted post-Covid patients (hospitalized or outpatient) by telephone at 44 and 223 days of follow-up. Persistence of symptoms was reported by 29.2% and 9.4% of patients, respectively, rates lower than ours (40%). The sequelae were more frequent in hospitalized patients in both periods. Olfactory symptoms were present at 4.2% and 0%, and abnormal sense of taste in 2.8% and 2.9%. Bianco et al. 29 also highlight that olfactory disorders are important symptoms in the acute and chronic phases.

Dysphonia in patients with Covid-19 is another symptom widely described. Some authors such as Lechien et al. 30 (26.8%) and Cantarella et al. (43.7%) 31 highlight a high prevalence. In our study, dysphonia was more prevalent in the acute phase of the disease (16%) than in the prolonged phase (1.8%), indicating a good recovery rate.

## Limitations of the Study

The limitations of this study were: the lack of otorhinolaryngological exams which would enable diagnostic confirmation of possible structural changes in the airway; difficulties in collecting data related to comorbidities, both in telephone contacts and when consulting medical records, making it difficult to establish a correlation between these variables and sequelae.

## Conclusion

Otorhinolaryngological symptoms were common in patients hospitalized for Covid-19, notably dyspnea, cough, nasal obstruction, and olfactory and taste disorders. Although with a favorable long-term course, 40% of patients remained symptomatic, notably with abnormal sense of smell, taste, and balance. The rates of sequelae were not related to the number of vaccine doses.
